# Mycobacteriophage Endolysins: Diverse and Modular Enzymes with Multiple Catalytic Activities

**DOI:** 10.1371/journal.pone.0034052

**Published:** 2012-03-28

**Authors:** Kimberly M. Payne, Graham F. Hatfull

**Affiliations:** Department of Biological Sciences, University of Pittsburgh, Pittsburgh, Pennsylvania, United States of America; French National Centre for Scientific Research - Université de Toulouse, France

## Abstract

The mycobacterial cell wall presents significant challenges to mycobacteriophages – viruses that infect mycobacterial hosts – because of its unusual structure containing a mycolic acid-rich mycobacterial outer membrane attached to an arabinogalactan layer that is in turn linked to the peptidoglycan. Although little is known about how mycobacteriophages circumvent these barriers during the process of infection, destroying it for lysis at the end of their lytic cycles requires an unusual set of functions. These include Lysin B proteins that cleave the linkage of mycolic acids to the arabinogalactan layer, chaperones required for endolysin delivery to peptidoglycan, holins that regulate lysis timing, and the endolysins (Lysin As) that hydrolyze peptidoglycan. Because mycobacterial peptidoglycan contains atypical features including 3→3 interpeptide linkages, it is not surprising that the mycobacteriophage endolysins also have non-canonical features. We present here a bioinformatic dissection of these lysins and show that they are highly diverse and extensively modular, with an impressive number of domain organizations. Most contain three domains with a novel N-terminal predicted peptidase, a centrally located amidase, muramidase, or transglycosylase, and a C-terminal putative cell wall binding domain.

## Introduction

Mycobacteriophages are viruses that infect mycobacterial hosts such as *Mycobacterium smegmatis* and *Mycobacterium tuberculosis* [1]. The complete sequences of more than 220 mycobacteriophage genomes have been determined from phages that are known to infect a single common host, *M. smegmatis* mc^2^155 [2], [3], [4]. These phages are highly genetically diverse and when grouped according to gross nucleotide sequence similarity they fall into 15 major clusters (A–O), many of which can be further divided into subclusters [2], [4], [5]. Genomes clustered together have nucleotide sequence similarity spanning more than 50% of their lengths and similar genome organizations; genomes within a subcluster have a greater average nucleotide identity than between subclusters [2]. An additional eight genomes are singletons and have no close relatives [2], [4]. In general, mycobacteriophage genomes are characteristically mosaic, with individual genes shared among otherwise unrelated genomes when compared at the amino acid sequence level [6]. In spite of this great genetic diversity, all of these are tailed phages containing double-stranded DNA (dsDNA) morphologically classified in the order *Caudovirales* [1].

As with all dsDNA-tailed bacteriophages, mycobacteriophages must ensure lysis of the host cell at the completion of the lytic cycle in order to release progeny phage particles [7], [8]. However, mycobacterial hosts have cell wall features that are distinct from most other bacterial hosts [9] and can present additional challenges to phage lysis [10], [11], [12]. The most notable of these features is the mycobacterial outer membrane, a mycolic acid-rich double layer that is covalently attached to a layer of arabinogalactan, which is turn in covalently linked to the peptidoglycan that surrounds the cytoplasmic membrane [13]. The presence of the mycobacterial outer membrane is highly unusual for bacteria classified within the Gram-positive *Actinomycetales*. In response to this, mycobacteriophages are atypical in encoding a lipolytic enzyme, Lysin B, an esterase that hydrolyzes the linkage of the mycolic acids to the peptidoglycan-arabinogalactan complex [10], [12], [14].

Aside from the unique mycobacterial outer membrane, the cytoplasmic membrane and peptidoglycan contribute to the integrity of the mycobacterial cell just as in both Gram-negative and Gram-positive bacteria [15], [16]. Phages employ a common system to destroy these structures that involves expression of an endolysin to cleave the peptidoglycan and a holin that permeabilizes the cell membrane to enable access of the endolysin to its substrate; holins also control the timing of lysis [17]. There is, however, considerable diversity among these components. For example, holin proteins act in a variety of ways, including the formation of holes in the membrane through which the endolysin can pass [17], as well as the pinholins that destabilize the membrane to activate endolysins carrying a Signal Arrest Release (SAR) domain at their N-terminus [18].

**Figure 1:**
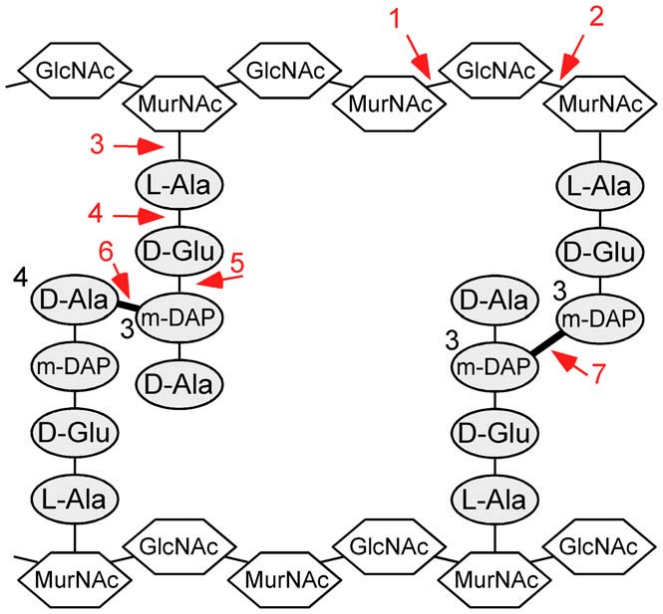
Schematic representation of mycobacterial peptidoglycan and generalized target bonds of peptidoglycan hydrolases. The type A1γ peptidoglycan of *M. tuberculosis* is proposed to contain the typical 4→3 interpeptide bridges between m-DAP and D-Ala but also 3→3 m-DAP to m-DAP bonds [30], [31]. There are seven positions within peptidoglycan where phage endolysins are known or are proposed to cleave (red arrows): 1, N-acetyl-β-D-muramidase (lysozyme, lytic transglycosylase); 2, N-acetyl-β-D-glucosaminidase; 3, N-acetylmuramoyl-L-alanine amidase; 4, L-alanoyl-D-glutamate (LD) endopeptidase; 5, γ-D-glutamyl-meso-diaminopimelic acid (DL) peptidase; 6, D-Ala-m-DAP (DD) endopeptidase; 7, m-DAP-m-DAP (LD) endopeptidase. Mycobacteriophage endolysins contain domains that are predicted to cleave all of these sites with the exception of position #2. The GH19, GH25, and TG domains cleave at position #1, Ami-2A and Ami-2B cleave at position #3, the N1 domain is predicted to cleave at position #4, the N5 position is predicted to cleave at #5, and the M23, N2 and N3 domains are predicted to cleave interpeptide bridges such as #6 and possibly #7. Peptide linkages to the central MurNAc residues, glycolylated muramic acid residues, and the amidation of D-Glu and m-DAP are not shown. GlcNAc, N-acetyl glucosamine; MurNAc, N-acetyl muramic acid; m-DAP, meso-diaminopimelic acid.

Phage endolysins are modular in their structures [19] and typically are composed of two components, an N-terminal catalytic domain and a C-terminal cell wall binding domain [7], [20], [21]. A variety of catalytic motifs are found including glycosidases that hydrolyze linkages of the aminosugar moieties and amidases and peptidases that attack the amide or peptide bonds of the cross-linking peptide or interpeptide bridges (Fig. 1) [20]. Peptidoglycan hydrolases are often specific to certain peptidoglycan crosslinking types and secondary modifications. Gram-positive bacteria contain few modifications to their glycan strands but also vary in their interpeptide bridges, and phages encode endolysins responsive to these differences [20]. One example is the D-Ala-Gly endopeptidase of phage phi11 that targets the penta-glycine interpeptide bridge in the peptidoglycan of *Staphylococcus aureus* [22].

A previously developed taxonomic system for classifying peptide bridges places mycobacteria in group A1γ, which also includes *Bacillus subtilis* and most Gram-negative bacteria including *Escherichia coli* [reviewed in [23]]. These have peptide bridges containing L-Ala, D-Glu, D-Ala, and meso-diaminopimelate (m-DAP) with interpeptide 4→3 D-Ala-m-DAP bridges (Fig. 1). The peptidoglycan of mycobacteria is distinctive in at least three respects. First, at least some of the muramic acid residues are N-glycolylated instead of N-acetylated, which is proposed to increase peptidoglycan integrity and resistance to lysozymes [24], [25], [26]. Second, the D-Glu and m-DAP in the peptide crosslink may be amidated [27]. And finally, the peptidoglycan is not only heavily crosslinked [∼80% relative to 30–50% in *Escherichia coli*, [28]] but at least in some growth states contains 3→3 m-DAP-m-DAP interpeptide bridges in addition to the more typical 4→3 interpeptide bonds (see Fig. 1) [29], [30], [31]. These 3→3 crosslinks are generated by an L-D-transpeptidase (MT2594) that is required for *M. tuberculosis* virulence [30], and are predominant in stationary phase cultures [31]. We noted previously that a derivative of mycobacteriophage TM4 from which a peptidoglycan hydrolase segment is removed from its tapemeasure protein shows a growth-state defect in infection relative to its parent phage [32], perhaps reflecting stationary phase-associated changes in peptidoglycan.

Mycobacteriophages encode endolysins (Lysin As) that are required for lysis at the end of lytic growth [10], [33]. However, several non-canonical features have been described. First, it has been shown that two products are expressed from the endolysin gene (*2*) of phage Ms6, the full length product of 384 residues, and a 241 residue protein derived from use of an internal translation initiation codon [34]; both products are required for the normal timing, progression and completion of host lysis. Secondly, delivery of the Ms6 endolysin (gp2) to its peptidoglycan target is dependent on a phage-encoded chaperone-like protein (gp1) that is required for lysis and acts in a holin-independent manner [35], [36]. Finally, prior bioinformatic analysis indicates the mosaic nature of mycobacteriophage endolysins [5], [37].

Phage-encoded endolysins have considerable potential as potent antimicrobial agents – or enzybiotics – against a number of Gram-positive bacterial pathogens [7], [21], [38], [39]. Unlike Gram-negative bacteria in which an outer membrane protects the peptidoglycan layer from external attack, phage endolysins have direct access to the peptidoglycan in Gram-positive bacteria. Although there is a substantial need for new anti-tuberculosis therapies – especially against multidrug resistant (MDR) and extensively drug resistant (XDR) strains – phage endolysins might be of limited use due to the mycobacterial outer membrane that likely prevents access to the peptidoglycan layer from without. Nonetheless, mycobacterial peptidoglycan and the enzymes that synthesize it remain prime targets for anti-tuberculosis drug development [30].

We describe here a bioinformatic analysis of the endolysins encoded by 224 sequenced mycobacteriophage genomes. They are highly modular in nature and most are composed of three domains: a C-terminal domain that is likely to be associated with binding to the cell wall, a central catalytic domain that is commonly a glycoside hydrolase (muramidase, transglycosylase) or amidase, and an N-terminal domain with putative peptidase activity. The prevalence of these peptidase domains, of which several variants have not been previously identified in phage endolysins, likely reflects the complexity of the peptidoglycan substrate with its high degree of crosslinking and its unusual interpeptide linkages. We also demonstrate hydrolysis and cell lysis by some of these endolysins including ones that lack known glycoside catalytic domains.

## Results

### Mycobacteriophage endolysins are diverse and modular

**Figure 2:**
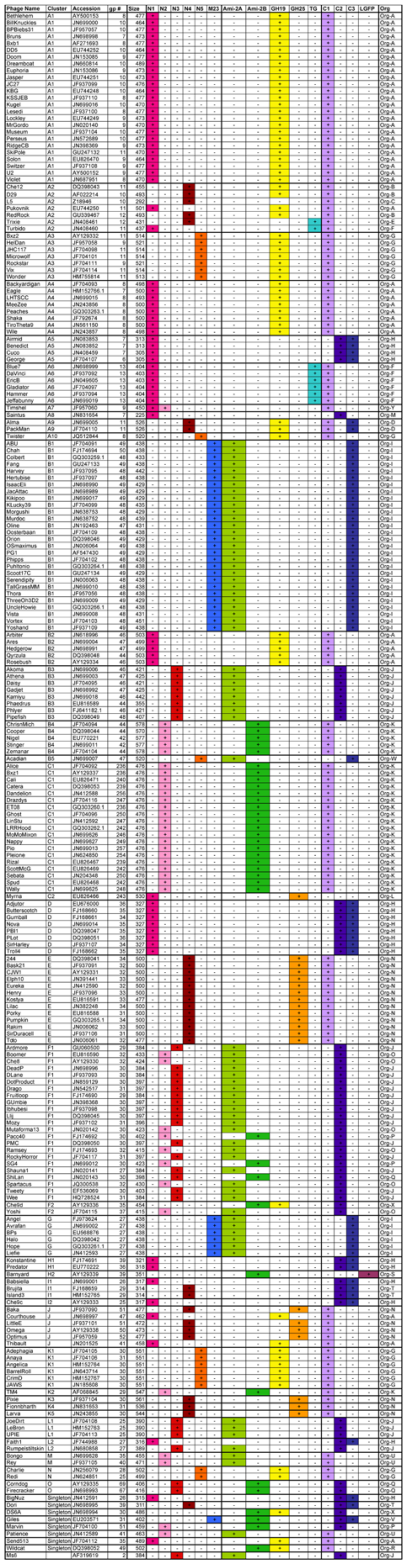
Domain organization of mycobacteriophage endolysins. Each of the mycobacteriophage endolysins is designated according to the presence (+) or absence (−) of each of three domain types: an N-terminal domain (N1, N2, N3, N4, N5, M23), a central catalytic domain (Ami-2A, Ami-2B, GH19, GH25, or TG), and a C-terminal domain (C1, C2, C3, or LGFP). Each is designated with an organizational type (Org) according to the domain content as listed in the rightmost column.

Phage endolysins typically are composed of two domains: an N-terminal domain with catalytic activity directed at hydrolysis of the peptidoglycan cell well, and a C-terminal cell wall binding domain [19], [21]. Prior comparative analysis of a small number of mycobacteriophage endolysins showed that they are similarly modular with shared segments (which we will refer to as domains) coupled to unrelated segments [5]. We have extended this analysis to a large number of mycobacteriophages (224), each of which is predicted to encode a single endolysin (Lysin A); none of these are interrupted by either introns or inteins. We have defined the numbers and types of modules present in these endolysins, as well as the variety of combinations that compose individual Lysin A proteins. The extent of each putative domain was deduced from BlastP and ClustalW searches (Table S1) and significance levels of e-values of 10^−5^ or 20% similarity respectively were used to define shared regions. A summary of the domains present in each of the endolysins is shown in Figure 2, and a summary of the domain features is shown in Table 1.

**Table 1:** Features of bioinformatically-defined Mycobacteriophage endolysin domains.

Domain	Putative Activity	M'phage example	HHPred match	E-value	Conserved domain
N1	L-Ala-D-Glu peptidase	Pukonvik gp11	*Listeria* phage A500 endolysin	2e-12	
N2	Hydrolase (M23B peptidase)	Bxz1 gp236	phi29 gp13 tail protein	0.0059	
N3	Hydrolase	Tweety gp30	phi29 gp13 tail protein	0.00027	
N4	Cysteine protease	L5 gp10	RipA (Rv1477)	1e-16	
N5	Cysteine protease	Bxz2 gp11	*B. anthracis* C39-like peptidase	6.6e-17	
M23	Peptidase	Giles gp31	V. cholerae zinc protease	8.9e-24	pfam01551
Ami-2A	N-acetyl-β-D-muramidase	BPs gp27	*E. coli* zinc amidase	1.8e-43	pfam01510
Ami-2B	N-acetyl-β-D-muramidase	Bxz1 gp236	B. subtilis XylA	1.2e-27	pfam01510
GH19	Glycoside hydrolase	Bxb1 gp8	*S. coelicolor* Chitinase G	3e-38	cd00325
GH25	Glycoside hydrolase	Kostya gp33	*B. anthracis* lysozyme	5.2e-43	cd06523
TG	N-acetyl-β-D-muramidase	Hammer gp13	*M. tuberculosis* RfpB	1.6e-23	pfam06737
C1	Cell wall binding?	Bxz2 gp11	None		
C2	Cell wall binding?	Corndog gp69	None		
C3/PGBD	Peptidoglycan binding domain	Giles gp31	*Pseudomonas* phage phiKZ Endolysin	3.6e-13	pfam01471
LGFP	Cell wall binding?	Barnyard gp39	None		pfam08310

**Figure 3:**
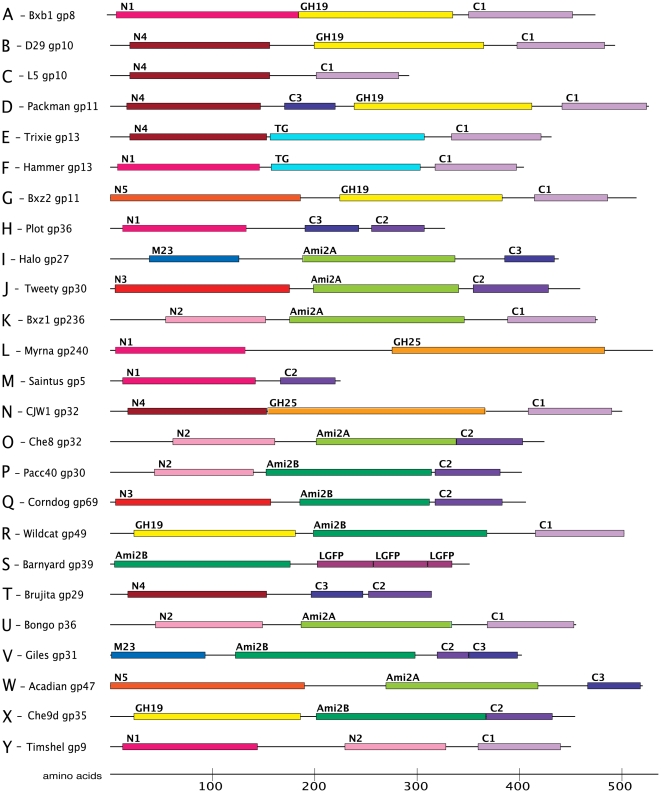
Modular organizations of mycobacteriophage endolysins. Through combinations of 15 domains and sequence elements, 26 different organizations are observed in the 240 mycobacteriophage Lysin As. Representative Lysin A structures shown for each organization. N1–N5 (red and pink shades) and C1–C3 (purple shades) are N-terminal and C-terminal regions, respectively. Predicted catalytic functions include Ami-2A and Ami-2B (amidases, green and aquamarine), GH19 (glycoside hydrolase family 19, yellow), GH25 (glycoside hydrolase family 25, orange), M23 (M23 family peptidase, blue), and TG (lytic transglycosylase, cyan). Also identified are the predicted binding motifs PGBD (peptidoglycan binding domain, gray) and LGFP repeats (rose).

The most striking feature of the mycobacteriophage endolysins is their amazing diversity, encompassing enormous variation and differing in size by more than two-fold (Figs. 2, 3). There are two key observations in regard to their organizational structures. The first is that most of them (∼90%) are composed of three conserved domains (Figs. 2, 3). This is in contrast to most other phage endolysins that contain only a catalytic domain and a cell wall binding domain, although we note that a few three-domain endolysins have been reported in other phages [22], [40], [41]. In the mycobacteriophage three-domain proteins, there is typically a C-terminal domain that likely corresponds functionally to the C-terminal cell wall binding domains of other phage endolysins, a central domain that contains a sequence motif known to be associated with peptidoglycan hydrolysis, and an N-terminal domain whose function is more obscure but appears to encode a variety of peptidases – these are discussed in greater detail below. There is considerable diversity among members of each domain type, such that pairs may share little more than 20% amino acid identity.

A second notable feature is the number and variety of the combinations in which these domains are assembled in the individual endolysins. With six possible types of N-terminal peptidase domain, five types of amidase/glycosidase domains, and four putative C-terminally positioned cell wall binding motifs, there are a least 120 possible combinations (assuming one of each of the three units), of which 25 are observed (Figs. 2, 3). These gene organizations are referred to as Org-A, Org-B etc (Figs. 2, 3). The vast majority of these lysins (211/224) are organized as an N-terminal putative peptidase domain, a central amidase, muramidase, or transglycosylase domain, and a C-terminal domain, and these constitute 15 of the 25 organizational types (Org-A, B, E, F, G, I, J, K, N, O, P, Q, U, V, and W; Fig. 3). Of the 10 organization types that do not follow this pattern (Org-C, D, H, L, M, R, S, T, X and Y, Fig. 3) all – with the notable exception of Myrna gp243 that apparently lacks a C-terminal cell-wall binding domain – contain at least one C-terminal motif, and some contain more than one. Curiously, the 24 lysins constituting Org-C, Org-H, Org-M, Org-S and Org-T lack a central catalytic domain with amidase, muramidase, glycosidase, or transglycosylase activity. The lysins of Org-H and Org-T have an N-terminal domain followed by two conserved C-terminal motifs. The five lysins of Org-R, Org-S, Org-X, and Org-Y lack a conserved N-terminal peptidase domain; Org-R and Org-X contain two versions of the non-peptidase catalytic domains, and in Timshel gp9 (Org-Y) there are two different types of N-terminal domain (Figs. 2, 3). In another non-canonical organization, Alma gp11 and Packman gp11 (Org-D) have a putative binding motif situated N-terminal to a glycoside hydrolase domain (Figs. 2, 3). The only Lysin A to follow the more common two domain organization seen in non-mycobacteriophage endolysins with N-terminal catalytic and C-terminal binding is Barnyard gp39 (Org-S), which has an amidase domain followed by a C-terminal domain with predicted cell wall binding motifs.

### Catalytic activities cleaving the peptidoglycan sugar backbone

Enzymatic activities that cleave the 1,4-β linkages between the N-acetylmuramic acid and N-acetylglucosamine sugars of the peptidoglycan backbone (Fig. 1, position #1) are common among bacteriophage endolysins [20] and are well-represented in the mycobacteriophage Lysin As. These are typically present as the central domains and three distinct sequence types are readily identifiable by BlastP and conserved domain searches (Table 1). Two of these are N-acetyl-β-D-muramidases belonging to the glycoside hydrolase families GH19 (cd00325) and GH25 (cd06523) [42] and are present in about 40% of these endolysins (Fig. 2). GH25 is a common muramidase among phage endolysins, but GH19 has not been characterized in phages. GH19 is classified as a chitinase that cleaves 1,4-β linkages between two N-acetylglucosamine sugars, a structure not typically seen in bacterial cell walls. Interestingly, except for *Mycobacterium*, *Corynebacterium*, and closely-related species, all GH19-containing endolysins belong to phages infecting Gram-negative hosts, primarily *Pseudomonas*.

The third enzymatic activity is a transglycosylase (TG) domain (pfam06737), also a member of the lysozyme_like superfamily (Table 1). There are relatively few examples of TG Lysin As and they are restricted to phages within Cluster A (Figs. 2, 3). In all eight examples the TG is the sole central catalytic domain and is flanked by conserved N-terminal and C-terminal domains. Interestingly, related transglycosylase domains are also found in the tape measure protein genes of mycobacteriophages [6] and the Hammer gp13 TG domain shares ∼70% amino acid sequence identity with a domain in the tape measure proteins of Cluster D phages; these also have similarity to the *M. tuberculosis* resuscitation promoting factor RpfA. Given the relative rarity of TG activity found thus far in mycobacteriophage and other phage endolysins, the resulting TG-containing endolysins are interesting variants, as most transglycosylase endolysins are found in phages infecting Gram-negative hosts, including the λ R endolysin [43] and the *Pseudomonas* phage φKZ144 lysin [44]; putative transglycosylase endolysins are also encoded in some staphylococcal and lactococcal phages. The three types of glycosidase domains, GH19, GH25, and TG, do not share sequence similarity recognizable after three iterations of PSI-Blast analysis and presumably derive from different evolutionary ancestors. We note that none of the mycobacteriophage endolysins appear to contain N-acetyl-β-D-glucosaminidase activities (see Fig. 1, position #2).

### Catalytic activities cleaving the N-acetylmuramoyl-L-alanine amide linkage

Amidases that cleave N-acetylmuramoyl-L-alanine linkages connecting the sugar backbone to the peptide crosslink (see Fig. 1, position #3) are common among phage endolysins and over 40% of the mycobacteriophage endolysins contain domains related to the amidase-2 conserved domain (pfam01510) (Figs. 2, 3). However, two features of these amidase domains emerge from comparative analysis. First, they span an enormous range of sequence diversity. For example, attempts to align pairs of more distantly related proteins – such as Bxz1 gp236 and Angel gp27 – show that they share no readily identifiable sequence similarity, even though a search for conserved domains identifies both as containing an amidase-2 domain (pfam01510) within the larger superfamily of PGRP peptidoglycan recognition proteins (cd06583)(Table 1). Second, phylogenetic analysis shows that the domains fall into two major clades, which we will refer to as amidase-2A (Ami-2A) and amidase-2B (Ami-2B) domains (Fig. S1). The Ami-2A domain is somewhat more prevalent than the Ami-2B domain (Figs. 2, 3).

### Putative peptidase domains in the mycobacteriophage endolysins

There are a number of potential peptidase targets within peptidoglycan, both within the pentapeptide chain and at the interpeptide crosslinks (Fig. 1, positions #4-7). Because the variations in peptidoglycan structure seen between bacterial species usually occur within these peptide components – including the 3→3 interpeptide bridges in *M. tuberculosis* [30] – endolysin peptidase activities are likely to be particularly informative about these structures.

The mycobacteriophage endolysins are replete with predicted peptidase domains, some of which have not been reported in other phage endolysins (Table 1). A total of six sequence variants are present: the M23 peptidase domain and N-terminal domains N1–N5 (Table 1), which show similarity to known peptidases but not to any currently identified conserved domains. The easiest to identify is the M23 peptidase domain (pfam01551) (Table 1). However, this is a component of just two domain organizations, Org-I and Org-V, which also have either an Ami-2A or an Ami-2B domain. In general, the M23 peptidase domain is observed in other phage endolysins, including in phages of *Thermus*, *Lactococcus*, *Entercococcus*, *Rhodococcus*, *Clostridium*, and *Lactobacillus*, and a putative prophage-encoded protein CwlP of *Bacillus subtilis* contains a similar domain and has been shown to function as a DD-endopeptidase cleaving 4→3 D-Ala–m-DAP interpeptide linkages (Fig. 1, position #6) [45]. Whether M23 peptidase domains also cleave 3→3 m-DAP–m-DAP linkages (Fig. 1, position #7) is not known.

Although the other peptidase motifs cannot be readily identified by conserved domain searches, significant matches to other peptidases were identified using HHPred searches [46], and these correspond to the N1–N5 domains (Table 1). Four different peptidase motifs are present. The N1 domain appears to encode an L-Ala–D-Glu peptidase activity (Fig. 1, position #4), which has been found in endolysins such as that of *Listeria* phage A500 [47] (Table 1) and coliphage T5 [48]. It is notable that while this activity is found in phages infecting both Gram-positive and Gram-negative bacteria, all the hosts have type A1γ peptidoglycan (Fig. 1), suggesting substrate and host specificity. The N2 and N3 domains are quite distinct from each other at the sequence level and none of the pairwise comparisons between N2 and N3 members shows more than 20% amino acid identity. However, HHPred searches show that both have significant similarity to the gp13 tail knob protein of phi29, which has a metalloprotease domain of the M23 family (Table 1). The N2 and N3 domains are therefore likely to be distant relatives of M23 peptidases. HHPred searches show that the N4 domain has similarity to several cell wall-associated mycobacterial proteins (Table 1) including a hypothetical cysteine protease of *M. avium* (MAP1204) and the resuscitation-promoting factor interacting protein of *M. tuberculosis*, RipA (Rv1477). The crystal structures of RipA and the related RipB protein show a peptidase active site that likely cleaves the 4→3 linkage between the D-Glu and m-DAP residues [28] (Fig. 1, position #5). To our knowledge this specificity has not been previously reported for phage endolysins. The N5 domain matches several structurally defined proteins by HHPred – including several cysteine proteases of *Staphylococcus aureus* – and these are generally defined as proteins of the large papain family (Table 1). The N5 domains of mycobacteriophage Lysin As are the first to be identified in phage endolysins to our knowledge.

### Identification of putative cell wall binding domains

With only a single exception (Myrna gp243), the putative endolysin catalytic domain is flanked on its C-terminal side by a second conserved domain, a position where other phage endolysins typically have a cell wall binding domain [21]. This domain has at least one of four distinct types of C-terminal sequence motifs in the Lysin A proteins, with the three more common termed C1, C2, and C3. The endolysins in organizations Org-D, Org-H, Org-I, Org-T, Org-V, and Org-W (Fig. 2) all contain a recognizable peptidoglycan binding domain (PGBD; pfam01471) that is part of a more extended shared region that we refer to as the C3 motif (Figs. 2, 3). Another motif, C2, is either found near C3 (Org-H, Org-T, and Org-V) or alone (Figs. 2, 3). The Alma gp11 and Packman gp11 (Org-D) lysins have both C1 and C3 motifs, but the C3 motif is located between the N-terminal and catalytic domains (Fig. 3). Neither C1 nor C2 motifs contain any recognizable conserved domains and HHPred searches were not informative. We speculate that these, however, may also be involved in cell wall recognition. Finally, Barnyard gp39 is alone among the mycobacteriophage endolysins in containing three sequential LGFP motifs (pfam08310; superfamily cl07065) (Figs. 2, 3). This 54-residue repeat is proposed to be involved in cell wall anchoring in *Corynebacterium* PS1 protein [49]. It is unusual to find this motif in phage endolysins and the only example we are aware of is in the *Tsukamurella* phage TPA2 [50].

### Combinatorial complexity of the Lysin A proteins

As described above, there are three types of segments in most of the mycobacteriophage endolysins, a central catalytic domain that targets either the sugar backbone or its linkage to peptide chain, a C-terminal domain that is presumed to be involved in cell wall binding, and an N-terminal domain with predicted peptidase activity. These are assembled into 25 distinct organizations (Org-A to Org-Y) (Figs. 2, 3). However, the four most prevalent organizations, Org-A, Org-I, Org-J, and Org-K (with 47, 38, 31 and 26 component members respectively) account for about 60% of all of the endolysins characterized here. In contrast, 11 of the organizations, Org-C, Org-D, Org-E, Org-L, Org-M, Org-R, Org-S, Org-V, Org-W, Org-X, and Org-Y each contain only a single component endolysin. Moreover, there is no strict correlation between the endolysin organization and the cluster or subcluster to which its genome belongs. For example, although Org-A is predominant among the Subcluster A1 phages, it is also present in Subclusters A2, A4, and B2, as well as in Cluster J and the singleton Send513 (Fig. 2). It is also notable that the eight Subcluster A2 phages reflect six different organizations (Org-A, Org-B, Org-C, Org-D, Org-E, and Org-F) (Fig. 2).

**Figure 4:**
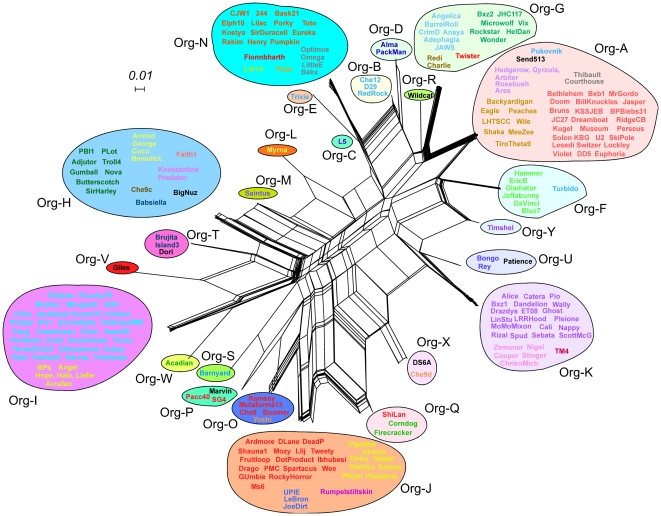
Network representation of mycobacteriophage Lysin A relationships. A matrix of the presence/absence of each of the 15 individual domains in the 224 genomes was analyzed using Splitstree and its NeighborNet function. Genomes within each of the 25 organizations (Org-A – Org-Y) are circled and individual genome names are colored according to cluster/subcluster. See Figure 2 for specific cluster/subcluster designations.

**Figure 5:**
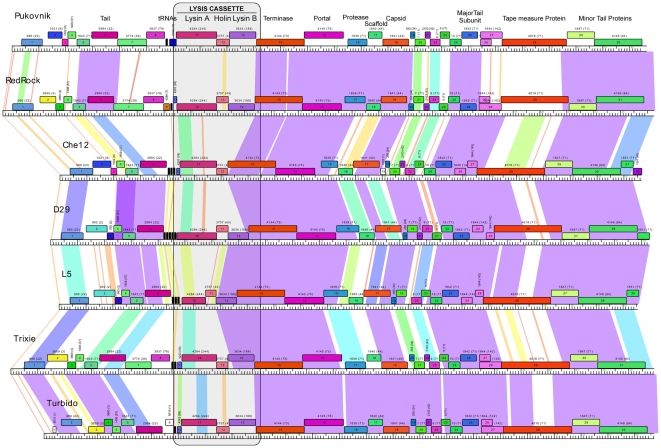
Comparison of the left parts of Cluster A2 genomes. Maps of the Subcluster A2 genomes were generated using the program Phamerator [58] and the left parts (approximately 20–25 kbp) are shown. Each of the predicted protein-coding genes is shown as a box with its color corresponding to its phamily designation [58]; the number of the phamily is shown above the gene with the number of phamily members in parentheses. Coloring of the regions between the genomes reflects the strength of pairwise nucleotide similarity, with the strength reflected according to the color spectrum (violet being the most similar, and the red the least similar). Note that there is higher divergence among the lysis genes and the flanking parts of the genome. An example of intragenic mosaicism reflected at the nucleotide sequence level is provided in the comparison of the L5, Trixie, and Turbido endolysins (Lysin As), reflecting domain organizations Org-C, Org-E, and Org-F respectively.

The relationships reflecting domain distributions in the 25 different organizations and the genomes and clusters they represent can be presented using the NeighborNet function in Splitstree [51] (Fig. 4). The complexity of the relationships reflects the pervasive modularity of these endolysins and the broad distribution of particular domains across genetically diverse genomes. The inclusion of genomes of several different clusters or subclusters within each organizational group – for example the genomes of six different cluster/subclusters (A5, D, H1, I1, I2, L2) and the singleton BigNuz – reflects the high rate of domain exchange relative to the genomes as a whole. Similarly, the endolysins of the seven A2 genomes have five different domain organizations (Fig. 2), and those of the 23 F1 genomes have four different domain organizations (Fig. 2). This is evident in comparison of the genome maps of the Cluster A2 phages (Fig. 5), where the divergence of the lysis cassette in general is considerably greater than the surrounding genes. Presumably there is a strong selective advantage to the exchange of these modules, perhaps in response to changes in host peptidoglycan that confer resistance to the action of these endolysins, or the ability to enhance the efficiency of lysis in an alternative host.

**Figure 6:**
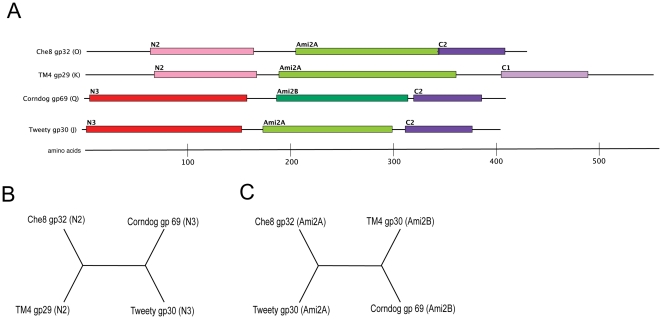
Recombination of domains between LysinAs. ClustalW alignments of four LysA proteins were used to construct a phylogenetic tree for the N-terminal and amidase domains. **A**. Proteins are identified by phage name, gp#, and Organizations. **B.** and **C.** Tree illustrating the most parsimonious phylogeny for the (**B**) N-terminal and (**C**) amidase domains. Bootstrap values are 100 for the division of recombination between the domains, and an SH test rejected alternate topologies (*P-*value <0.001). Branch lengths do not represent evolutionary distance.

**Figure 7:**
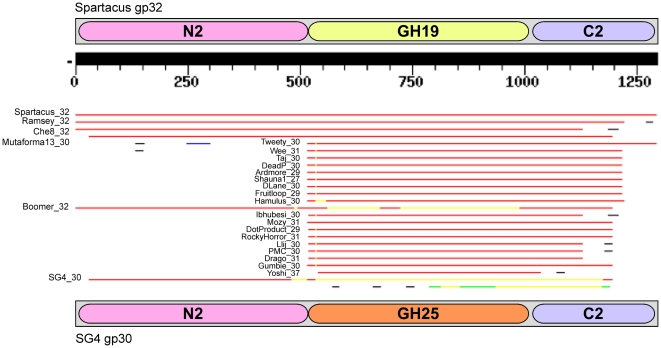
Domain swap between Spartacus gp32 and SG4 gp30. The locations of the three domains in the two proteins are shown, flanking the BLASTP output generating by a search using Spartacus gp32 as the query.

Che8 gp32, Corndog gp69, TM4 gp29, and Tweety gp30 represent examples of four distinct organizations (Org-J, Org-K, Org-O, Org-Q) (Fig. 2) and the domains have distinct phylogenies (Fig. 6). For example, the N-terminal domains N2 and N3 have a phylogeny (Fig. 6B) that is distinct from that of the Ami-2A and Ami-2B domains (Fig. 6C). TM4 (Subcluster K2) and Corndog (singleton) are very different at the gross genomic level compared to Che8 and Tweety (both of which are in Subcluster F1) and recombination between phages of these groups presumably gave rise to the observed modularity of their Lysin A proteins. In a second example, a BlastN search of the mycobacteriophage Spartacus gene *32* sequence against a database of mycobacterial genomes identifies three full-length homologues (Ramsey_*32*, Che8_*32*, and Mutaforma13_30) that share the same organization (Org-O), as well other genes that have partial matches (Fig. 7). Specifically, there are 18 matches (Tweety_30, Wee_31 etc) corresponding to Org-J (Figs. 2, 7), reflecting the main difference between Org-O and Org-J being the N-terminal domain (which have N2 and N3 respectively). One gene, SG4_30, matches only the 5′ end of the Spartacus_32 gene, indicating the presence of a different central catalytic domain (i.e. Ami-2B rather than Ami-2A; see Fig. 2). Boomer gp32 shows a strong match at both the 5′and 3′ ends of the genes, but a poor match in the middle. In this instance Boomer gp32 has an Ami-2A domain as in Spartacus gp32, but it is more highly diverged such that DNA sequence similarity is obscured. These patterns likely reflect the site of recombination events that have constructed these chimeric proteins, although it is interesting to note that the exchanges may introduce a distinctly different motif, such as the Ami-2B in SG4, or just a divergent copy of the same motif as in Boomer gp32.

### Mycobacteriophage endolysins departing from the three-domain organization

The observation that some organizations lack the central catalytic domain (Org-C, Org-H, Org-M, and Org-T) is consistent with the interpretation that the N-terminal domains are important for peptidoglycan hydrolysis. These are all relatively small proteins (<325 aa) and include the smallest of the mycobacteriophage endolysins, L5 gp10 and Saintus gp5, which are 292 and 225 residues respectively. Although the N-terminal peptidases are abundantly represented, there are three organizations (Org-R, Org-S and Org-X) that lack an N-terminal peptidase domain, suggesting that it is not absolutely required. However, two of these (Org-R and Org-S) are notable in that they contain two central catalytic domains, with a GH19 in an N-terminal position to an Ami-2A or Ami-2B. There are also two examples of four-domain proteins. Timshel gp9 (Org-Y) appears to contain two N-terminal domains, N1 and N2, in addition to GH19 and C1 domains. Packman and Alma gp11 (Org-D) contain only one N-terminal domain (N4) but have two possible “C-terminal” domains, with C3 and C1 motifs flanking its central GH19 domain (Fig. 3).

### Expression of secondary gene products

Catalão *et al* [34] recently showed that the Ms6 *lysA* gene (*2*) – whose primary product gp2 shares 99% amino acid identity with Fruitloop gp29 – encodes a second gene product (LysA_271_) resulting from translation initiation at codon 144. They also predicted that Lysin As with similar organizations can be expressed similarly, as with Boomer gp32, Che8 gp32 and Ramsey gp32. Although this is a reasonable extension from the Ms6 gp2 observations, we note that the putative translation initiation signals are predicted to be weak, raising the question as to whether these play a similar *in vivo* role. We note, however, as reported previously [10], that when the Lysin A proteins of Corndog (gp69), Bxz1 (gp236) and Che8 (gp32) are expressed in *E. coli* and the preparations analyzed by zymography with lyophilized *Micrococcus luteus* all show products smaller than the full length protein that can hydrolyze peptidoglycan. Internal gene starts may therefore indeed be a common phenomenon with *lysA* genes.

**Figure 8:**
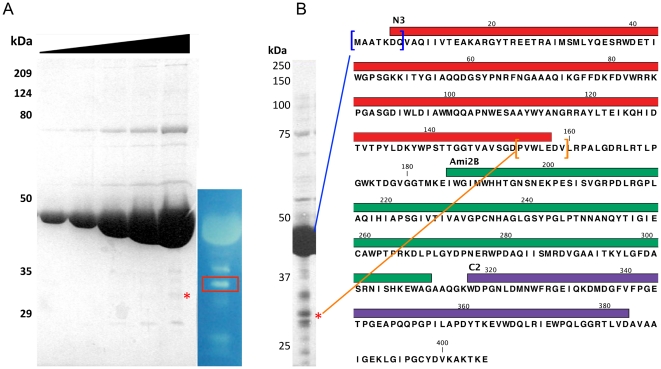
Identification of Corndog gp69 fragment with increased lytic activity. **A.** Left, SDS PAGE with increasing amounts of Corndog gp69 sample illustrating the minute amount of protein (red asterisk) corresponding to one of the bands of high activity seen in the zymogram on the right (red box). **B.** Highly concentrated sample of Corndog gp69 from which a sample was taken (red asterisk) and submitted to Edman degradation. The control sequencing of the large band matched the N-terminal sequence of Corndog gp69 (blue brackets), while the smaller fragment corresponded to a sequence 153 aa C-terminal to the start of Corndog gp69 (orange brackets).

To extend this we analyzed the Corndog gp69 expression further (Fig. 8). The protein is expressed well in *E. coli* with the primary product a protein of 439 amino acids and we confirmed that the start side we predicted bioinformatically [6] is the one used by N-terminal sequencing of the protein (Fig. 8B). The zymogram of the same preparation showed hydrolytic activity of full-length protein, but several smaller proteins also show activity (Fig. 8A). As there is so little protein visible by Coomassie Blue staining, these fragments must have a specific activity that is substantially greater than the full-length protein. We obtained sufficient material to get N-terminal sequence information for the most active of these products and found it to be consistent with a start site at codon 147 (Fig. 8B). This is at a similar location to where the second start site is located in Ms6 *lysA*. While we cannot eliminate the possibility that the smaller product is generated by proteolysis, its apparent high specific activity of the truncated Ami2B-C2 form of Corndog gp69 suggests that the N3 N-terminal domain was inhibiting catalysis in this zymogram assay. Although observations of activity using zymograms are only poorly quantitative, we note a similar effect is observed with the Ms6 Lysin A protein [34].

### Apparent holin-independent lysis by some Lysin A proteins

Expression of phage endolysins alone in bacteria does not typically result in cellular lysis because the enzyme fails to reach its catalytic target in the cell wall, and activation of catalysis is triggered by the holin that provides a timely passage of the enzyme through the cell membrane [17]. Indeed, we observe this with Giles gp31 that we expressed to complement the lysis defect in a Giles Δ*31* deletion mutant (unpublished observations). However, in the course of these experiments we noted that expression of the D29 endolysin (gp10) appeared to lead to cell death, even though the putative holin gene (*11*) was excluded from the expression clone. We therefore tested to what extent this occurred when other mycobacteriophage endolysins are expressed in *M. smegmatis*.

**Figure 9:**
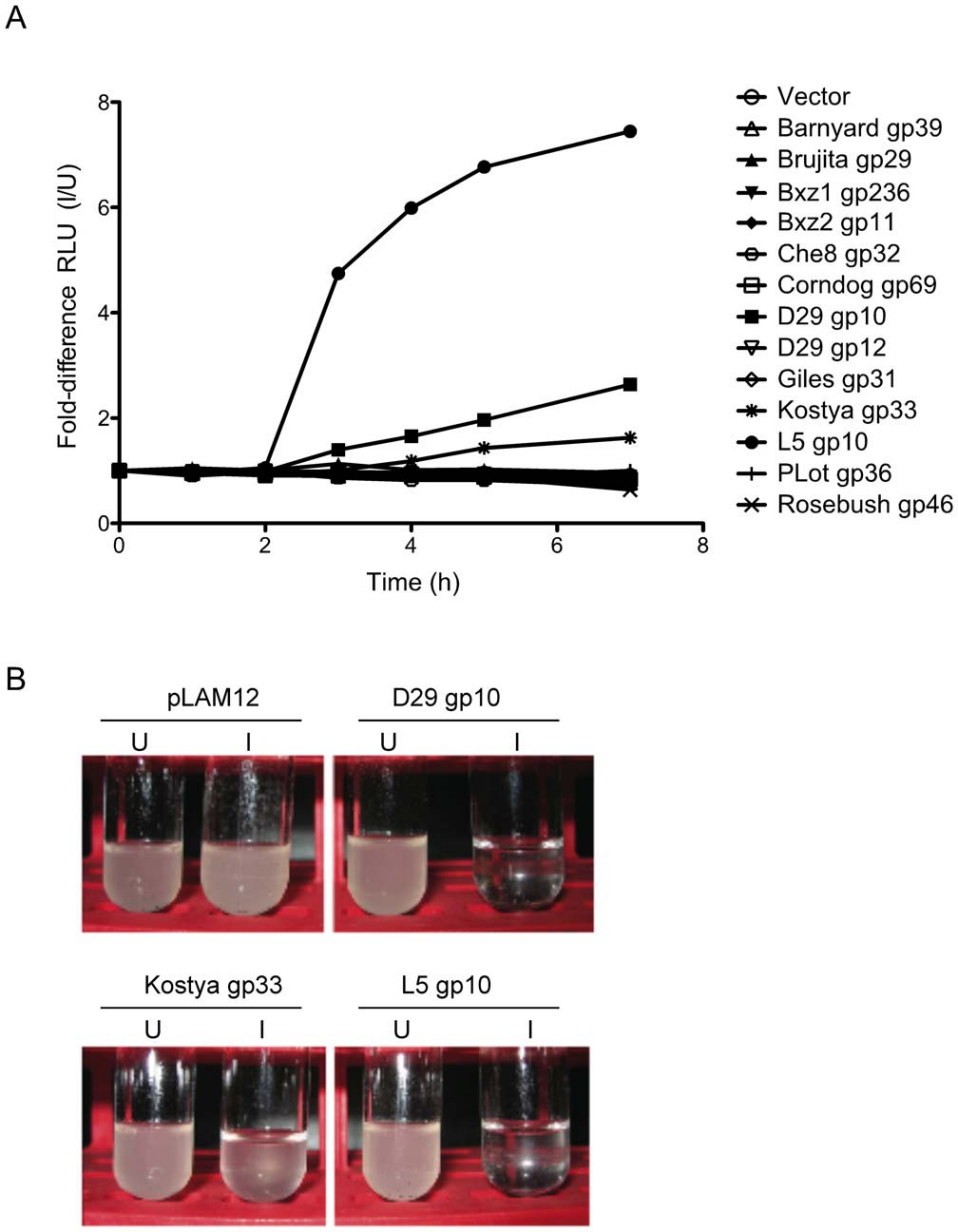
ATP release upon induction of lysin expression. Cultures of *M. smegmatis* mc2155 carrying pLAM12-based plasmids with different lysins were split and half were induced with 0.2% acetamide. **A.** ATP release was measured for 7 hours and the fold-difference between induced and uninduced ATP release calculated for each lysin and plotted versus the time. **B.** After more than 16 hours of induction several of the cultures had lysed completely.

Of the 12 different endolysins (and one Lysin B, D29 gp12) that we tested (Fig. S2), we observed expression of proteins of the anticipated sizes in at least nine of them (Fig. S3); the notable failures were Barnyard gp39, Corndog gp69, and L5 gp10. However, in all the constructs where expression was seen the protein was only poorly soluble, and most of it remained in the pellet after clarification of the sample (Fig. S3). When we monitored cell lysis using an ATP-release assay, most of the Lysin A proteins had no effect on cell growth, as expected. Interestingly, three of the proteins did show an increase in ATP-release (Fig. 9A) and yielded visibly lysed cultures after 16 h (Fig. 9B). With both D29 gp10 and Kostya gp33 the extent of ATP release was modest, although after extended induction substantial clearing of the culture was observed with D29 gp10. In contrast, expression of L5 gp10 caused a dramatic rise in ATP-release three hours after induction and lysis appeared to be complete after 7 hours (Fig. 9). These three endolysins share N4 and C1 domains (Fig. 2). D29 gp10 and Kostya gp33 are predicted to have different lysozyme activities – GH19 and GH25, respectively – but L5 gp10 is comprised solely of an N4 domain and a C1 motif. As C1 was found in many of the non-active Lysin As (Fig. S2), we consider them unlikely to be responsible for the lytic activity, but it is possible that they can influence it. Brujita gp29 also has an N4 domain with no other catalytic activity followed by a C-terminal domain with C2 and C3 motifs, but despite its robust expression (Fig. S3), did not show any evidence of lysis (Fig. 9A). Because of the possibility of a second holin gene wholly embedded within the *lysA* gene, we searched for the presence of potential transmembrane domains within all six possible translation frames of L5 gene *10*; none were found and a second holin gene seems an unlikely possibility. We also note that none of the Lysin A proteins contain N-terminal SAR domains, including L5 gp10.

## Discussion

We have described here a detailed dissection of the endolysins encoded by mycobacteriophages. The complexity of these endolysins is truly amazing, with each being composed of exchangeable domains assembled into a large variety of organizations. Although a total of 15 different domains have been identified, these fall into three main types, and the majority of the endolysins have one of 4–6 domains from each of the three menus, in a common order – an N-terminal peptidase, a central non-peptidase catalytic domain, and a C-terminal motif presumed to be involved in cell wall binding. A subset of the endolysins departs from this and either lack one of the three units, or has multiple copies of them.

The prevalence and variety of the peptidase motifs in the mycobacteriophage endolysins is unusual and likely reflects the complexity of the cell wall of their mycobacterial hosts. A complication in the interpretation of this is that the range of potential hosts for the sequenced mycobacteriophages is not known. They all share a common host, *M. smegmatis* mc^2^155, but there are several lines of evidence suggesting that they may also infect other hosts, and that the broad diversity of the phages reflects a range of overlapping host preferences [1]. Host preferences may also correspond to cluster/subcluster designations, and we note for example that the subset of phages that also infect *M. tuberculosis* are restricted to the Cluster K and Subcluster A2 phages [2], [3]. In further support of this, there is a close correlation between cluster/subcluster designations and genome GC% content, perhaps also reflecting different host preferences [1]. In light of this, the complexity of the endolysins and especially the variety of peptidase motifs may reflect differences in peptidoglycan linkages within lineages of the *Actinomycetales*.

Although nearly all of the mycobacteriophages encode both an endolysin and a Lysin B, there are a few examples in which the Lysin B gene is absent. These include Che12 and Packman (Subcluster A2, Org-B), Arbiter, Ares, Hedgerow, Qyrzula, and Rosebush (Subcluster B2, Org-A), Myrna (Subcluster C2, Org-L), and Charlie and Redi (Cluster N, Org-G). However, there is little or no correlation between the types of endolysins encoded by these (Fig. 4), and thus there is no evidence that a particular organization of endolysin compensates for the lack of a Lysin B.

The intragenic mosaicism of these endolysins is especially striking and represents a microcosm of the relationships among the phage genomes as a whole [6]. The simple interpretation is that these are undergoing a high rate of variation relative to the genomes as a whole, with domains being actively exchanged between phages. Presumably this is facilitated by horizontal genetic exchange mediated by illegitimate recombination, as proposed for the genomics as a whole [37]; there is no evidence of short conserved sequences between the domains that might promote exchange by homologous recombination. The notable manifestation of this is the observation that phage genomes grouped into a cluster or a subcluster according to their gross nucleotide relationships often include a variety of different endolysin domain organizations.

The apparent holin-independent mycobacterial lysis by the L5 endolysin is noteworthy in that the activity demonstrates that enzyme can potently act on the cell wall even though it is predicted to contain only an N-terminal peptidase domain and a presumed C-terminal cell wall binding domain. Although lysis occurs efficiently in the absence of the holin gene, it is plausible that a high level of protein expression and interaction with the cell membrane could cause sufficient destabilization to enable access of the enzyme to its peptidoglycan substrate. It seems unlikely that lysis during an L5 infection would be entirely holin independent, because the genome encodes a good holin candidate (gp11) with two predicted membrane-spanning domains, although a holin-defective mutant of L5 is required to resolve this question.

The mycobacteriophage endolysins have potential utility as antimycobacterial agents just as enzybiotics have been successfully developed for other gram-positive bacteria [21]. However, access of the mycobacteriophage endolysins to their peptidoglycan substrate is thwarted by the mycolic acid rich mycobacterial outer membrane, and it is unclear if this could be promoted by inclusion of the Lysin B enzymes [10], [14]. Moreover, the diversity of the endolysins described here presents the possibility of using these in combination such that a highly effective variety of enzymatic activities could be provided with just a small number of individual proteins. This notwithstanding, the observation that efficient lysis can be mediated by intracellular expression of some of these endolysins may of practical utility in a variety of settings.

## Methods and Methods

### Bioinformatic Analyses

Lysin A protein sequences were subjected to BLASTp and PSI-BLAST to identify homologous regions of sequence from non-redundant sequences at NCBI (blast.ncbi.nlm.nih.gov). Multiple sequence alignments were performed using the ClustalW algorithm executed using the CLC Bio Main Workbench (v. 4.1.2) software package, which was also used for general sequence analysis and alignments. Distinct regions of homology were identified and grouped into domains with a threshold of e-value <1×10^−5^ and a ClustalW identity >20%. Domains were attributed potential functions based on hits to conserved domain families from the Pfam [52], COG [53], and InterPro [54] databases. Phylogenetic analyses used PHYML 3.0 [55] to generate maximum likelihood phylogenetic trees based on ClustalW scores with 100 rounds of bootstrapping and an SH test. Phylogenetic trees were drawn using NJPlot. A search for any transmembrane domains or signal sequences was performed using TMHMM Server 2.0 [56] and SignalP 3.0 [57], respectively. HHPred searches were performed using the server at http://toolkit.tuebingen.mpg.de/hhpred [46]. Splitstree 4.0 [51] was used to display the relationships of the endolysins according to their domain composition. An input nexus file was generated from an excel file containing presence/absence information for each domain in each endolysin using the converter Janus (J. Lawrence, unpublished information). Phamerator was used to generate genome maps using the default parameters [58].

### Bacterial strains and growth

All *M. smegmatis* mc^2^155 strains were cultured in Middlebrook 7H9 medium or grown on Middlebrook 7H10 agar supplemented with 10% Albumin Dextrose Complex (ADC), 0.2% succinate, 0.05% Tween-80, 1 mM Ca2Cl, carbenicillin (50 mg/ml), cyclohexamide (10 mg/ml) and kanamycin (20 mg/ml) as required. *E. coli* was grown in L-broth (LB) supplemented with carbenicillin (50 mg/ml) and kanamycin (20 mg/ml) as needed, with *E. coli* GC5 cells (Stratagene) used for cloning and *E. coli* BL21(DE3) cells (Stratagene) for protein overexpression.

### Cloning and purification of Lysin As

Cloning and purification of Lysin As for expression in *E. coli* and *M. smegmatis* was performed as described previously [10]. Briefly, *lysA*s were cloned with primers bearing NdeI and HindIII restriction sites into pET21a (Novagen) for IPTG-induced expression of C-terminally His-tagged protein in *E. coli* BL21(DE3) cells, then purified by affinity chromatography using TALON Co^2+^ resin; after two washes with 10 and 20 mM imidazole, protein was eluted with 120 mM imidazole. Eluted fractions were concentrated using Vivaspin concentration columns (molecular weight cut-off 10 kDa; Sartorius) followed by dialysis against storage buffer (50 mM Tris pH 8.0, 50 mM NaCl, 50% glycerol) and stored at −20°C.

### Zymograms

Zymograms were performed as described previously [32] by incorporation of 0.2% lyophilized *Micrococcus luteus* cells as a source of peptidoglycan into the gel matrix. Zymograms were developed by renaturation overnight at 37°C in 25 mM Tris (pH 7.5), 1% Triton X-100 and 0.1 mM ZnSO4, then stained with 0.5% methylene blue with 0.01% KOH before destaining with water.

### Endogenous expression of Lysin As in *M. smegmatis*

For expression in *M. smegmatis* mc^2^155, *lysA*s were cloned into pLAM12 [59] for acetamide-induced expression. Upon reaching an OD_600 nm_ of 0.4–0.6, *M. smegmatis* with a pLAM12-based *lysA* construct was induced with 0.2% acetamide and incubated with shaking at 37°C. Protein expression was assessed by taking a 1 ml sample prior to induction and at the end of the 7 h period, separating the cells into a pellet and supernatant fractions by centrifugation, lysing the pellet by sonication, and loading amounts equalized with respect to the culture OD_600_ at the time of sampling onto an SDS PAGE gel. The procedure for the ATP release assay is similar to that described in Payne et al. (2009). Briefly, after reaching log-phase *M. smegmatis* was induced and diluted to an OD_600_ of 0.03 so that readings were within the measuring range of the luminometer. Every hour 100 µl samples were taken, combined with 100 µl of ENLITEN rLuciferase/Luciferin reagent (Promega), and luminescence recorded for a 10 second interval in a Monolight 2010 luminometer. The results were reported as the fold-difference in ATP-release compared to a control culture containing pLAM12. Growth inhibition and lysis was also tracked visually by following the OD_600_ of induced cultures incubating with shaking at 37°C for up to 36 hours as compared to uninduced controls.

## Supporting Information

Figure S1 **Phylogenetic tree of Lysin A amidase domains.** A neighbor-joining tree based on a ClustalW alignment of amidase domains found in Lysin As. Numbers indicate distance between nodes.
(PDF)

Figure S2 **Cloned Lysin As for expression in** ***M. smegmatis.*** The above 12 Lysin As were cloned into the acetamide-inducible pLAM12 vector. They were chosen to represent the diversity of domains, with each domain represented at least once. Organizations are listed to the right of the Lysin A name in parentheses.
(PDF)

Figure S3 **Expression profiles of lysins induced in** ***M. smegmatis*** **.** The above samples are from the cultures used in the ATP assay described in A.3.2. One milliliter samples were taken from induced and uninduced cultures with pLAM12 vector control, one of 12 Lysin As, or one Lysin B (D29 gp10). These were sonicated, centrifuged at 14,000 rpm to produce pellet and supernatant fractions, and separated on SDS PAGE gels. Red asterisks mark identifiable expressed protein. Red question marks indicate instances of uncertain protein expression.
(PDF)

Table S1 **Coordinates of mycobacteriophage endolysin domains.**
(PDF)
