# Supplementary figures and images for: Mycobacteriophage Endolysins: Diverse and Modular Enzymes with Multiple Catalytic Activities
Source: PLoS One. 2012 Mar 28;7(3):e34052. doi: 10.1371/journal.pone.0034052 (PMC3314691; doi:10.1371/journal.pone.0034052)

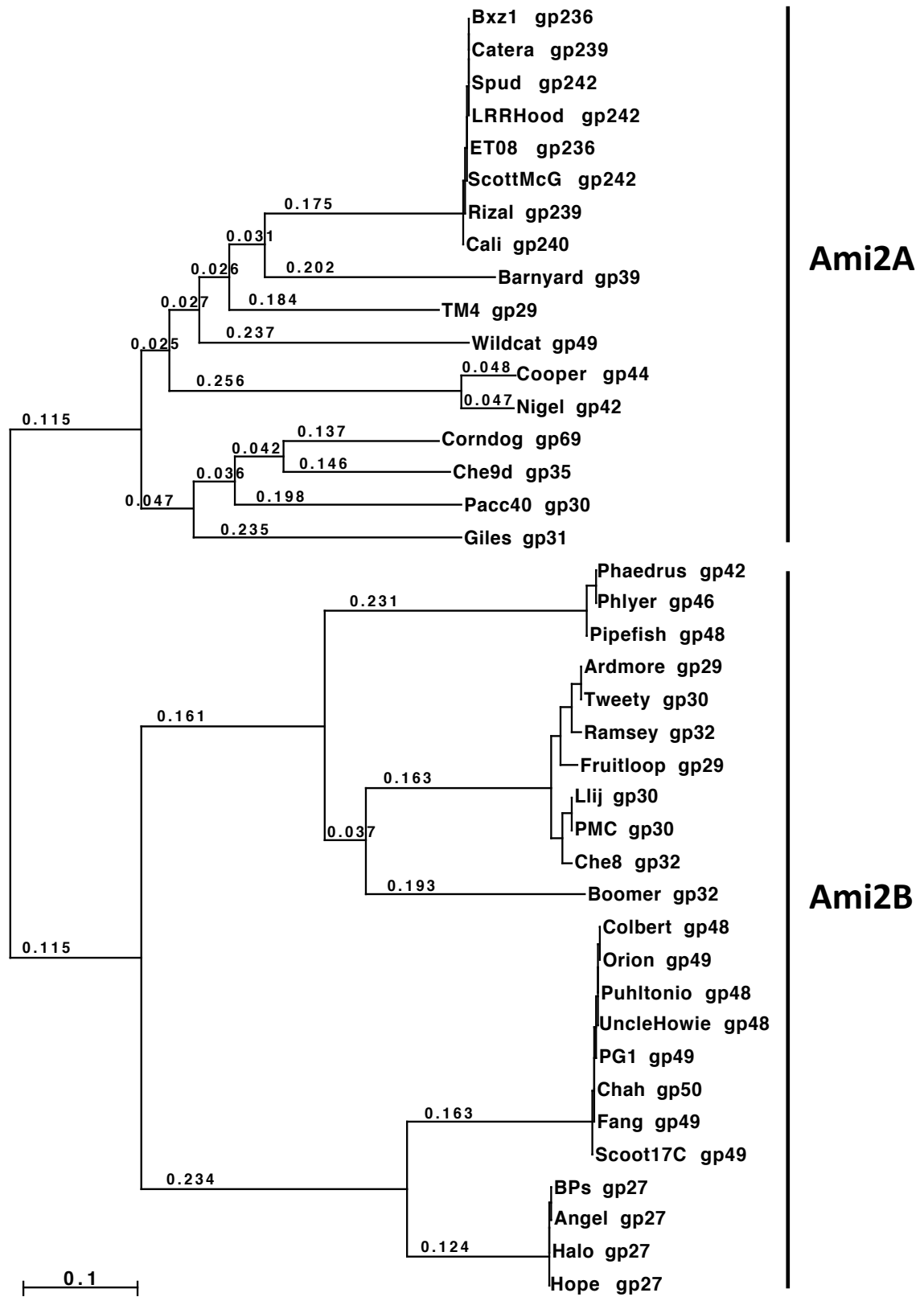

Figure S1

Supplement: Figure S1 — Phylogenetic tree of Lysin A amidase domains. A neighbor-joining tree based on a ClustalW alignment of amidase domains found in Lysin As. Numbers indicate distance between nodes. (PDF) [file pone.0034052.s001.pdf]

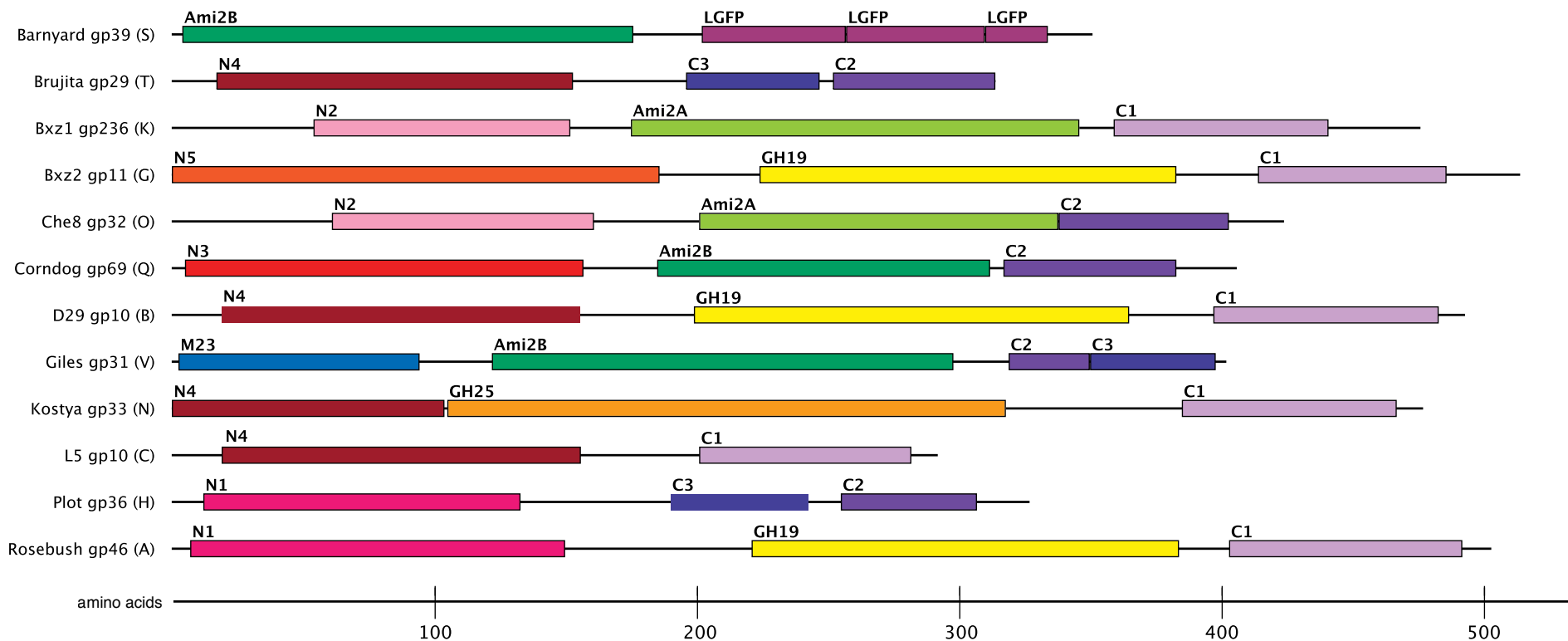

Figure S2

Supplement: Figure S2 — Cloned Lysin As for expression in M. smegmatis. The above 12 Lysin As were cloned into the acetamide-inducible pLAM12 vector. They were chosen to represent the diversity of domains, with each domain represented at least once. Organizations are listed to the right of the Lysin A name in parentheses. (PDF) [file pone.0034052.s002.pdf]

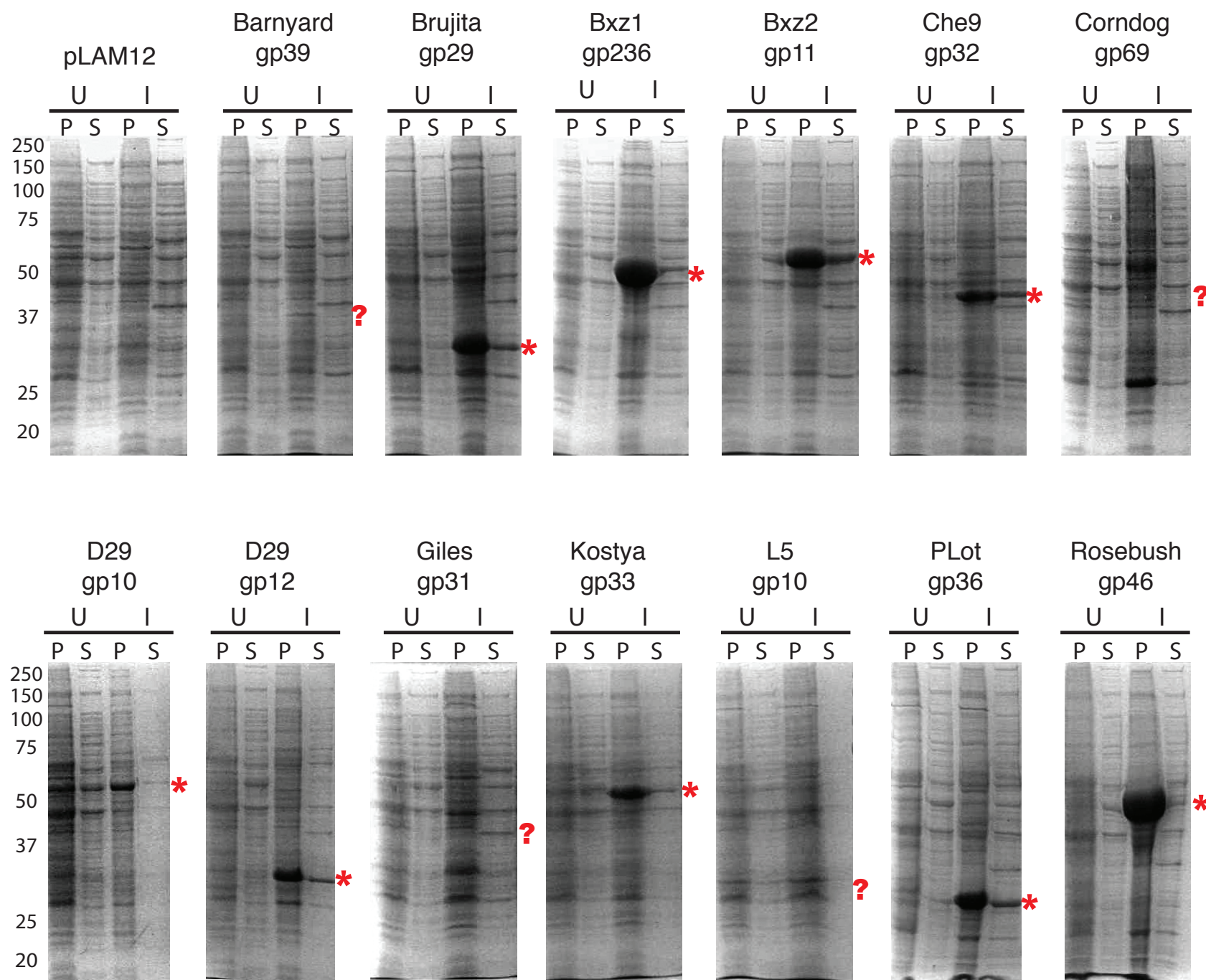

Figure S3

Supplement: Figure S3 — Expression profiles of lysins induced in M. smegmatis . The above samples are from the cultures used in the ATP assay described in A.3.2. One milliliter samples were taken from induced and uninduced cultures with pLAM12 vector control, one of 12 Lysin As, or one Lysin B (D29 gp10). These were sonicated, centrifuged at 14,000 rpm to produce pellet and supernatant fractions, and separated on SDS PAGE gels. Red asterisks mark identifiable expressed protein. Red question marks indicate instances of uncertain protein expression. (PDF) [file pone.0034052.s003.pdf]
