# Supplementary material for: Mycobacteriophage Endolysins: Diverse and Modular Enzymes with Multiple Catalytic Activities
Source: PLoS One. 2012 Mar 28;7(3):e34052. doi: 10.1371/journal.pone.0034052 (PMC3314691; doi:10.1371/journal.pone.0034052)
Supplement: Table S1 — Coordinates of mycobacteriophage endolysin domains. (PDF) [file pone.0034052.s004.pdf]

Table S1. Coordinates of mycobacteriophage endolysin domains

Phage Name	Cluster	Accession	gp #	Size	Org	N1	N2	N3	N4	N5	M23	Ami-2A	Ami-2B	GH19	GH25	TG	C1	C2	C3	LGFP
Bethlehem	A1	AY500153	8	477	Org-A	12-136								198-347			382-464			
BillKnuckles	A1	JN699000	10	464	Org-A	4-130								185-334			369-451			
BPBiebs31	A1	JF957057	10	477	Org-A	12-136								198-347			382-464			
Bruns	A1	JN698998	7	473	Org-A	13-142								194-343			378-460			
Bxb1	A1	AF271693	8	477	Org-A	12-136								198-347			382-464			
DD5	A1	EU744252	10	464	Org-A	4-130								185-334			369-451			
Doom	A1	JN153085	9	477	Org-A	12-136								198-347			382-464			
Dreamboat	A1	JN660814	10	489	Org-A	11-182								183-351			364-465			
Euphoria	A1	JN153086	9	473	Org-A	13-142								194-343			378-460			
Jasper	A1	EU744251	10	473	Org-A	13-142								194-343			378-460			
JC27	A1	JF937099	10	476	Org-A	12-136								198-347			382-464			
KBG	A1	EU744248	10	464	Org-A	4-130								185-334			369-451			
KSSJEB	A1	JF937110	8	477	Org-A	12-136								198-347			382-464			
Kugel	A1	JN699016	10	470	Org-A	12-136								191-340			375-457			
Lesedi	A1	JF937100	8	477	Org-A	12-136								198-347			382-464			
Lockley	A1	EU744249	9	473	Org-A	13-142								194-343			378-460			
MrGordo	A1	JN020140	9	470	Org-A	12-136								191-340			375-457			
Museum	A1	JF937104	10	477	Org-A	12-136								198-347			382-464			
Perseus	A1	JN572689	10	477	Org-A	12-136								198-347			382-464			
RidgeCB	A1	JN398369	9	473	Org-A	13-142								194-343			378-460			
SkiPole	A1	GU247132	11	470	Org-A	12-136								191-340			375-457			
Solon	A1	EU826470	9	464	Org-A	4-130								185-334			369-451			
Switzer	A1	JF937108	9	477	Org-A	12-136								198-347			382-464			
U2	A1	AY500152	9	477	Org-A	12-136								198-347			382-464			
Violet	A1	JN687951	8	470	Org-A	12-136								191-340			375-457			
Che12	A2	DQ398043	11	455	Org-B				20-154					198-347			369-448			
D29	A2	AF022214	10	493	Org-B				20-156					200-365			398-483			
L5	A2	Z18946	10	292	Org-C				20-156								202-282			
Pukovnik	A2	EU744250	11	501	Org-A	20-156								207-365			406-493			
RedRock	A2	GU339467	12	493	Org-B				20-156					200-365			396-483			
Trixie	A2	JN408461	12	431	Org-E				20-153							157-307	334-421			
Turbido	A2	JN408460	11	437	Org-F	13-141										158-307	342-426			
Bxz2	A3	AY129332	11	514	Org-G					1-186				225-383			415-486			
HelDan	A3	JF957058	9	521	Org-G					1-186				224-387			419-484			
JHC117	A3	JF704098	11	514	Org-G					1-186				225-383			415-486			
Microwolf	A3	JF704101	11	514	Org-G					1-186				225-383			415-486			
Rockstar	A3	JF704111	9	521	Org-G					1-186				224-387			419-506			
Vix	A3	JF704114	11	514	Org-G					1-189				225-383			415-486			
Wonder	A3	HM755814	11	513	Org-G					1-185				224-382			414-485			
Backyardigan	A4	JF704093	8	498	Org-A	13-141								205-363			404-490			
Eagle	A4	HM152766.1	7	500	Org-A	13-141								204-362			404-490			
LHTSCC	A4	JN699015	8	493	Org-A	13-143								207-365			396-483			
MeeZee	A4	JN243856	8	500	Org-A	13-141								204-362			404-490			
Peaches	A4	GQ303263.1	8	500	Org-A	13-141								204-362			404-490			
Shaka	A4	JF792674	8	500	Org-A	13-141								204-362			404-490			
TiroTheta9	A4	JN561150	8	500	Org-A	13-141								204-362			404-490			
Wile	A4	JN243857	8	498	Org-A	13-141								205-363			404-490			
Airmid	A5	JN083853	7	313	Org-H	21-140												253-313	192-244	
Benedict	A5	JN083852	7	313	Org-H	21-140												253-313	192-244	
Cuco	A5	JN408459	7	305	Org-H	13-132												245-305	185-236	
George	A5	JF704107	6	305	Org-H	13-132												245-305	185-236	
Blue7	A6	JN698999	13	404	Org-F	8-146										158-302	317-396			
DaVinci	A6	JF937092	13	403	Org-F	8-146										158-302	317-396			
EricB	A6	JN049605	13	403	Org-F	8-146										158-302	317-396			
Gladiator	A6	JF704097	13	404	Org-F	8-146										158-303	318-397			
Hammer	A6	JF937094	13	404	Org-F	8-146										158-303	318-397			
Jeffabunny	A6	JN699019	13	404	Org-F	8-146										158-303	318-397			
Timshel	A7	JF957060	9	450	Org-Y	13-144	230-328										360-440			
Saintus	A8	JN831654	7	225	Org-M	13-142												167-220		
Alma	A9	JN699005	11	526	Org-D				17-147					239-412			442-524		171-220	
PackMan	A9	JF704110	11	526	Org-D				17-147					239-412			442-524		171-220	
Twister	A10		8	520	Org-G					1-184				224-389			424-520			
ABU	B1	JF704091	49	438	Org-I						39-126	188-336							385-436	
Chah	B1	FJ174694	50	438	Org-I						41-128	188-336							385-436	
Colbert	B1	GQ303259.1	48	433	Org-I						32-119	181-333							380-431	
Fang	B1	GU247133	49	438	Org-I						39-126	188-336							385-436	
Harvey	B1	JF937095	48	442	Org-I						38-125	189-337							386-437	
Hertubise	B1	JF937097	48	438	Org-I						39-126	188-336							385-436	
IsaacEli	B1	JN698990	49	429	Org-I						34-121	181-329							378-429	
JacAttac	B1	JN698989	49	429	Org-I						32-119	179-327							376-427	
Kikipoo	B1	JN699017	49	429	Org-I						34-121	181-329							378-429	
KLucky39	B1	JF704099	48	435	Org-I						34-121	185-333							382-433	
Morgushi	B1	JN638753	48	429	Org-I						34-121	181-329							378-429	
Murdoc	B1	JN638752	48	439	Org-I						39-126	188-336							385-436	
Oline	B1	JN192463	47	431	Org-I						34-121	181-329							378-429	
Oosterbaan	B1	JF704109	48	438	Org-I						39-126	188-336							385-436	
Orion	B1	DQ398046	49	429	Org-I						32-119	179-327							376-427	
OSmaximus	B1	JN006064	49	438	Org-I						39-126	188-336							385-436	
PG1	B1	AF547430	49	429	Org-I						32-119	179-327							376-427	
Phipps	B1	JF704102	48	438	Org-I						39-126	188-336							385-436	
Puhltonio	B1	GQ303264.1	48	438	Org-I						39-126	188-336							385-436	
Scoot17C	B1	GU247134	49	429	Org-I						32-119	179-327							376-427	
Serendipity	B1	JN006063	48	438	Org-I						41-128	188-336							385-436	
TallGrassMM	B1	JN699010	48	438	Org-I						39-126	188-336							385-436	
Thora	B1	JF957056	48	438	Org-I						41-128	188-336							385-436	
ThreeOh3D2	B1	JN699009	49	429	Org-I						34-121	181-329							378-429	
UncleHowie	B1	GQ303266.1	48	438	Org-I						41-128	188-336							385-436	
Vista	B1	JN699008	48	431	Org-I						34-121	181-329							378-429	
Vortex	B1	JF704103	48	451	Org-I						38-125	185-333							382-433	
Yoshand	B1	JF937109	49	438	Org-I						39-126	188-336							385-436	
Arbiter	B2	JN618996	46	503	Org-A	8-150								222-384			404-492			

Ares	B2	JN699004	47	499	Org-A	4-146								218-380			400-488			
Hedgerow	B2	JN698991	47	499	Org-A	4-146								218-380			400-487			
Qyrzula	B2	DQ398048	44	503	Org-A	8-150								222-384			404-492			
Rosebush	B2	AY129334	46	503	Org-A	8-150								222-384			404-492			
Akoma	B3	JN699006	46	421	Org-J			29-182				204-330						351-414		
Athena	B3	JN699003	47	425	Org-J			29-199				208-333						347-425		
Daisy	B3	JF704095	46	421	Org-J			29-182				204-330						351-414		
Gadjet	B3	JN698992	47	425	Org-J			33-199				208-333						341-425		
Kamiyu	B3	JN699018	46	442	Org-J			50-203				225-350						456-442		
Phaedrus	B3	EU816589	44	355	Org-J			2-129				138-263						269-355		
Phlyer	B3	FJ641182.1	46	421	Org-J			29-182				204-330						351-414		
Pipefish	B3	DQ398049	48	407	Org-J			15-168				190-316						334-400		
ChrisnMich	B4	JF704094	44	578	Org-K		67-182						212-395				437-576			
Cooper	B4	DQ398044	44	570	Org-K		67-182						212-395				437-568			
Nigel	B4	EU770221	42	577	Org-K		67-182						212-395				436-575			
Stinger	B4	JN699011	42	577	Org-K		67-182						212-395				436-575			
Zemanar	B4	JF704104	44	578	Org-K		67-182						212-395				437-550			
Acadian	B5	JN699007	47	520	Org-W					1-190		270-418								
Alice	C1	JF704092	236	476	Org-K		55-152						176-346				389-474			
Bxz1	C1	AY129337	236	476	Org-K		55-152						176-346				389-474			
Cali	C1	EU826471	240	476	Org-K		55-152						176-346				389-474			
Catera	C1	DQ398053	239	476	Org-K		55-152						176-346				389-474			
Dandelion	C1	JN412588	256	476	Org-K		55-152						176-346				389-474			
Drazdys	C1	JF704116	247	476	Org-K		55-152						176-346				389-474			
ET08	C1	GQ303260.1	236	476	Org-K		55-152						176-346				389-474			
Ghost	C1	JF704096	250	476	Org-K		55-152						176-346				389-474			
LinStu	C1	JN412592	247	476	Org-K		55-152						176-346				389-474			
LRRHood	C1	GQ303262.1	242	476	Org-K		55-152						176-346				389-474			
MoMoMixon	C1	JN699626	246	476	Org-K		55-152						176-346				389-474			
Nappy	C1	JN699627	249	476	Org-K		55-152						176-346				389-474			
Pio	C1	JN699013	257	476	Org-K		55-152						176-346				389-474			
Pleione	C1	JN624850	254	476	Org-K		55-152						176-346				389-474			
Rizal	C1	EU826467	239	476	Org-K		55-152						176-346				389-474			
ScottMcG	C1	EU826469	242	476	Org-K		55-152						176-346				389-474			
Sebata	C1	JN204348	250	476	Org-K		55-152						176-346				389-474			
Spud	C1	EU826468	242	476	Org-K		55-152						176-346				389-474			
Wally	C1	JN699625	248	476	Org-K		55-152						176-346				389-474			
Myrna	C2	EU826466	243	530	Org-L	6-132									276-483					
Adjutor	D	EU676000	36	327	Org-H	13-133												256-307	191-243	
Butterscotch	D	FJ168660	35	327	Org-H	13-133												256-307	191-243	
Gumball	D	FJ168661	34	327	Org-H	13-133												256-307	191-243	
Nova	D	JN699014	35	327	Org-H	13-133												256-307	191-243	
PBI1	D	DQ398047	35	327	Org-H	13-133												256-307	191-243	
PLot	D	DQ398051	36	327	Org-H	13-133												256-307	191-243	
SirHarley	D	JF937107	34	327	Org-H	13-133												256-307	191-243	
Troll4	D	FJ168662	35	327	Org-H	13-133												256-307	191-243	
244	E	DQ398041	34	500	Org-N				18-153						155-367		409-490			
Bask21	E	JF937091	32	500	Org-N				18-153						155-367		409-490			
CJW1	E	AY129331	32	500	Org-N				18-153						155-367		409-490			
Elph10	E	JN391441	33	500	Org-N				18-153						155-367		409-490			
Eureka	E	JN412590	32	500	Org-N				18-153						155-367		409-490			
Henry	E	JF937096	33	500	Org-N				18-153						155-367		409-490			
Kostya	E	EU816591	33	477	Org-N				2-104						106-318		360-441			
Lilac	E	JN382248	34	500	Org-N				18-153						155-367		409-490			
Porky	E	EU816588	31	500	Org-N				18-153						155-367		409-490			
Pumpkin	E	GQ303265.1	34	500	Org-N				18-153						155-367		409-490			
Rakim	E	JN006062	33	500	Org-N				18-153						155-367		409-490			
SirDuracell	E	JF937106	31	500	Org-N				18-153						155-367		409-490			
Toto	E	JN006061	32	477	Org-N				2-130						132-344		386-467			
Ardmore	F1	GU060500	29	384	Org-J			5-154				175-299						313-376		
Boomer	F1	EU816590	32	433	Org-O		62-161					208-347						348-412		
Che8	F1	AY129330	32	424	Org-O		62-161					202-338						339-403		
DeadP	F1	JN698996	30	384	Org-J			5-154				175-299						313-376		
DLane	F1	JF937093	30	384	Org-J			5-154				175-299						313-376		
DotProduct	F1	JN859129	30	397	Org-J			5-154				175-299						313-376		
Drago	F1	JN542517	31	397	Org-J			5-154				175-299						313-376		
Fruitloop	F1	FJ174690	29	384	Org-J			5-154				175-299						313-376		
GUmbie	F1	JN398368	30	397	Org-J			5-154				175-299						313-376		
Ibhubesi	F1	JF937098	30	397	Org-J			5-154				175-299						313-376		
Llij	F1	DQ398045	30	397	Org-J			5-154				175-299						313-376		
Mozy	F1	JF937102	31	396	Org-J			5-153				174-298						312-375		
Mutaforma13	F1	JN020142	30	423	Org-O		60-161					201-337						338-399		
Pacc40	F1	FJ174692	30	402	Org-P		44-140						153-314					318-381		
PMC	F1	DQ398050	30	397	Org-J			5-154				175-299						312-376		
Ramsey	F1	FJ174693	32	415	Org-O		62-161					202-338						339-400		
RockyHorror	F1	JF704117	31	397	Org-J			5-167				175-298						311-386		
SG4	F1	JN699012	30	423	Org-P		61-160						178-352					341-398		
Shauna1	F1	JN020141	27	384	Org-J			5-154				175-299						313-376		
ShiLan	F1	JN020143	30	398	Org-Q			5-154					184-308					314-377		
Spartacus	F1	JQ300538	32	430	Org-O		1-161					203-325						337-403		
Tweety	F1	EF536069	30	403	Org-J			5-154				175-299						312-376		
Wee	F1	HQ728524	31	384	Org-J			5-154				175-299						313-376		
Che9d	F2	AY129336	35	454	Org-X								202-379	32-187				380-454		
Yoshi	F2	JF704115	37	415	Org-O		44-141					184-320						321-403		
Angel	G	FJ973624	27	438	Org-I						39-126	189-337							386-344	
Avrafan	G	JN699002	27	438	Org-I						39-126	189-337							386-344	
BPs	G	EU568876	27	438	Org-I						39-126	189-337							386-344	
Halo	G	DQ398042	27	438	Org-I						39-126	189-337							386-344	
Hope	G	GQ303261.1	27	438	Org-I						39-126	189-337							386-344	
Liefie	G	JN412593	27	438	Org-I						39-126	189-337							386-344	
Konstantine	H1	FJ174691	39	321	Org-H	13-134												259-321	194-246	
Predator	H1	EU770222	36	318	Org-H	13-134												255-318	190-242	
Barnyard	H2	AY129339	39	351	Org-S								5-176							203-334

Babsiella	I1	JN699001	26	317	Org-H	2-95												220-269	157-207	
Brujita	I1	FJ168659	29	314	Org-T				18-153									253-314	197-247	
Island3	I1	HM152765	29	314	Org-T				18-153									253-314	197-247	
Che9c	I2	AY129333	25	317	Org-H	4-132												257-317	194-244	
Baka	J	JF937090	51	477	Org-N				17-154						154-344		409-466			
Courthouse	J	JN698997	47	462	Org-A	12-185								186-357			370-459			
LittleE	J	JF937101	51	472	Org-N				18-147						149-339		404-461			
Omega	J	AY129338	50	473	Org-N				18-147						149-339		404-461			
Optimus	J	JF957059	52	477	Org-N				17-154						154-344		409-466			
Thibault	J	JN201525	41	458	Org-A	13-137								200-358			389-449			
Adephagia	K1	JF704105	30	551	Org-G					1-177				219-385			427-495			
Anaya	K1	JF704106	31	551	Org-G					1-177				219-385			427-495			
Angelica	K1	HM152764	30	551	Org-G					1-177				219-385			427-495			
BarrelRoll	K1	JN643714	30	551	Org-G					1-177				219-385			427-495			
CrimD	K1	HM152767	30	551	Org-G					1-177				219-385			427-495			
JAWS	K1	JN185608	30	551	Org-G					1-177				219-385			427-495			
TM4	K2	AF068845	29	547	Org-K		67-165						187-357				401-484			
Pixie	K3	JF937104	30	561	Org-N				17-147						149-361		413-550			
Fionnbharth	K4	JN831653	31	536	Org-N				25-158						173-352		353-492			
Larva	K5	JN243855	30	544	Org-N				18-147						149-361		415-532			
JoeDirt	L1	JF704108	25	390	Org-J			60-158				180-303						317-381		
LeBron	L1	HM152763	25	390	Org-J			14-158				180-303						317-381		
UPIE	L1	JF704113	25	390	Org-J			14-158				180-303						317-381		
Faith1	L2	JF744988	27	316	Org-H	12-140												260-303	195-249	
Rumpelstiltskin	L2	JN680858	27	389	Org-J			14-157				179-302						316-380		
Bongo	M	JN699628	35	455	Org-U		45-149					188-334					369-453			
Rey	M	JF937105	40	471	Org-U		67-165					205-351					385-469			
Charlie	N	JN256079	28	502	Org-G					1-178				223-385			409-490			
Redi	N	JN624851	25	499	Org-G					1-178				221-383			406-487			
Corndog	O	AY129335	69	406	Org-Q			6-157					186-312					318-383		
Firecracker	O	JN698993	67	416	Org-Q			6-157					186-312					318-383		
BigNuz	Singleton	JN412591	26	315	Org-H	13-134												258-315	193-245	
Dori	Singleton	JN698995	39	311	Org-T				18-153									253-311	197-247	
DS6A	Singleton	JN698994	30	486	Org-X								211-386	42-195			387-486			
Giles	Singleton	EU203571	31	402	Org-V						2-93		123-298					320-350	351-398	
Marvin	Singleton	JF704100	51	459	Org-P		67-182						235-355					363-428		
Patience	Singleton	JN412589	41	463	Org-U		49-151					203-344					379-455			
Send513	Singleton	JF704112	35	489	Org-A	4-142								214-360			401-481			
Wildcat	Singleton	DQ398052	49	502	Org-R								199-368	24-181			416-502			
Ms6		AF319619	2	384	Org-J			5-154				175-299						313-376		
